# Femoral bifurcation and bilateral tibial hemimelia: case report

**DOI:** 10.11604/pamj.2018.30.99.11969

**Published:** 2018-06-05

**Authors:** Joshua Ondari, James Kinyanjui, Paul Miano, Edward Sang, Ezekiel Oburu, Michael Maru

**Affiliations:** 1 Orthopaedic Surgeon, Nakuru County Hospital, Nakuru, Kenya; 2 Orthopaedic Surgeon, St Marys Hospital, Nakuru, Kenya; 3 Orthopaedic Surgeon, PCEA Kikuyu Rehabilitation and Orthopaedic Hospital, Kikuyu, Kenya; 4 Orthopaedic Surgeon and Lecturer, University of Nairobi, Nairobi, Kenya; 5 Orthopedic Surgeon and Clinical Services Manager, PCEA Kikuyu Rehabilitation and Orthopaedic Hospital, Kikuyu, Kenya

**Keywords:** Tibia hemimelia, bifid femur, Gollop-Wolfgang complex

## Abstract

Femoral bifurcation and tibial hemimelia are rare anomalies described as a variant of Gollop-Wolfgang complex. This article presents a case of Gollop-Wolfgang complex without hand ectrodactyly. A 5-year old patient presented with bilateral tibial hemimelia and left femoral bifurcation. The patient's left limb lacked knee extensor mechanism, disarticulation was done. The right leg which had Jones type 2 tibia hemimelia was treated with tibiofibular synostosis. Currently patient is ambulant with prosthesis on the left limb and ankle foot orthosis on the right. In the absence of proximal tibial anlage, especially in patients with femoral bifurcation, the knee should be disarticulated. Tibiofibular synostosis is a good choice in the presence of a proximal tibial anlage with good quadriceps function.

## Introduction

Tibial hemimelia, either partial or complete, is a very rare anomaly with an incidence of one in one million live births [1]. Congenital absence of the tibia with ipsilateral bifid femur is even rarer. The two conditions may occur independently in isolation [2] but more commonly, they co-exist and are accompanied by other congenital anomalies of the limbs or other parts of the body [3]. In 1980, Gollop et al [4] described two brothers with ectrodactyly of one hand, unilateral femoral bifurcation, bilateral absence of tibia and monodactyly of the feet. In 1984, Wolfgang [5] reported a single case of tibia hemimelia with ipsilateral femoral bifurcation and contralateral diastasis of the tibia. In 1986, the eponym 'Gollop-Wolfgang complex' was introduced by Lurie and Ilyina [6] as they concluded that the association of hand ectrodactyly and femoral bifurcation is not coincidental. The term 'Gollop-Wolfgang complex' is also used in patients with bifid femur and tibial agenesis without hand ectrodactyly [7]. The etiology of Gollop-Wolfgang complex is postulated to be an error in the complex genetic control of limb development but exact cause remains unclear [8]. Gollop-Wolfgang complex is listed as a rare disease by the United States Office of Rare Diseases [ORD] of the National Institute of Health [NIH]. Our literature search revealed about 200 cases reported worldwide without a similar case report in the region. A case of Gollop-Wolfgang complex without hand electrodactyl is presented, with follow-up of 3 years after initiation of surgical treatment. Informed consent was obtained from the mother for publication of this case report

## Patient and observation

**Figure 1:**
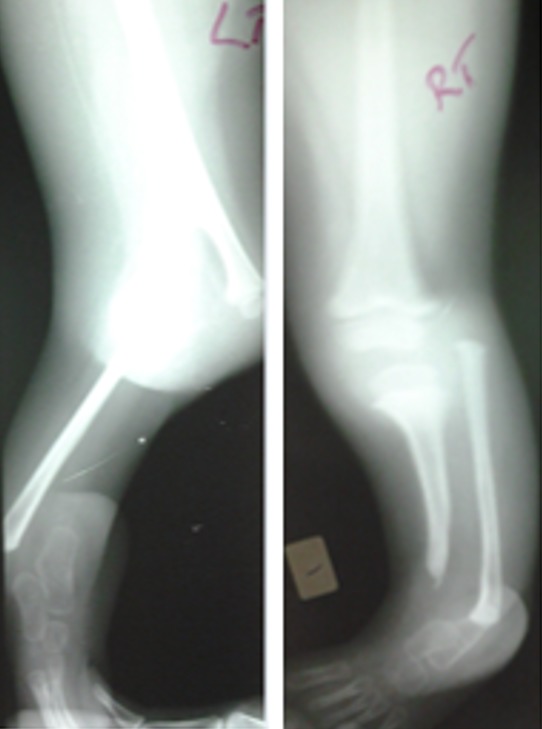
Right and left radiographs of femur and tibia/fibula

**Figure 2:**
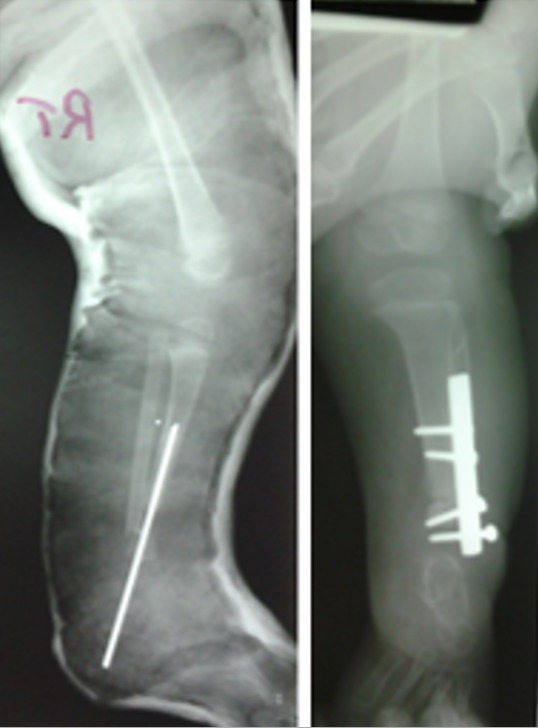
Radiographs after tibiofibular synostosis and rash rod insertion and after exchange to plating

**Figure 3:**
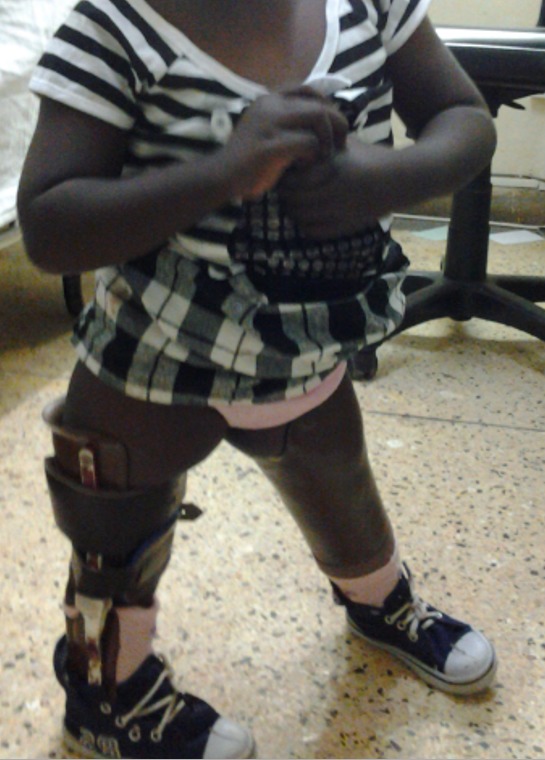
Child ambulating with left lower limb prosthesis and right ankle foot orthosis

FWM, a 5-year-old female, was seen at P.C.E.A Kikuyu Rehabilitation Hospital in February 2013 with congenital deformities of both lower limbs. She was the third born and only living child of a Kenyan family. She was born at term through cesarean section. Mother had threatened abortion at three months gestation during which she was diagnosed with hypertension but no record of medication used. She did not report use of any teratogenic drugs, alcohol consumption or smoking and no history of infection or diabetes. Her first born died soon after birth due to severe asphyxia and second born a week after birth due to severe pneumonia but no limb or visceral anomalies reported. No parental consanguinity was present and family history was negative for birth defects. The child had a normal level of intelligence and physical examination revealed the following abnormalities: *left lower limb*: the distal end of the thigh was widened with triangular appearance; palpation revealed bifurcation of distal part of the femur. The terminal portion of the medial branch was easily felt under the skin. The lateral branch articulated with a fibula suggesting some form of knee joint. There was a 120^0^ knee flexion contracture with 30^o^ painless range of motion and lack of active extension. The leg was shortened with a medial curvature and an equinovarus foot. The left hip joint was normal; *right lower limb*: the leg was shortened with severe equinovarus deformity of the feet. She had active knee extension with full range of motion. The hip was normal. Radiographs revealed left distal bifid femur with complete tibia deficiency and right partial distal tibia deficiency (Figure 1). Both upper limbs were normal and on further evaluation the child did not have any visceral or congenital cardiac anomalies. In February 2013, right tibiofibular synostosis was done and fixed with a rash rod with medial soft tissue release of the foot. She later underwent rash pin removal and plating due to nonunion. Plate was removed in November 2014 with good union (Figure 2). In August 2014 left knee disarticulation was done with excision of the medial branch of bifid femur. Three years later she was doing well ambulating with left limb prosthesis and right ankle foot orthosis (Figure 3).

## Discussion

Femoral bifurcation is a rare defect. It was first described by Ehrlich in 1885 in a case associated with tibial hypoplasia. Since then there have been sporadic reports of the same defect [9-11]. According to the theory of Lewin and Opitz [12], growth of the lower limb is under control of two developmental fields; the tibia and fibula. The tibial developmental field controls development of the distal femur, tibia and hallux. Thus a defect in this field results in distal femur duplication, tibia agenesis and preaxial polydactyly or ectrodactyly. The fibula developmental field controls the development of the fibula, lateral rays of the foot, lateral knee ligaments, proximal femur, acetabulum and pubic bones. A defect in this field results in fibular hypoplasia, ectrodactyly, proximal focal femoral deficiency and deficiency of lateral knee ligament. There appears to be a strong relationship between the development of the fibula and tibial fields as reported in literature [13]. In our case, the tibial developmental field was defective. There are three classification systems of tibial deficiency to guide treatment; Kalamchi and Dawe, Jones and the new weber classification. Jones classification which is the most commonly utilized has divided it into four types:- type 1: the tibia cannot be seen on radiographs at birth. In subtype 1a, the tibia is completely absent and ossific nucleus of the distal femoral epiphysis is hypoplastic. In subtype 1b, the proximal part of tibia is present, but unossified at birth, hence appears absent on plain radiograph. In this type, there is normal ossification of the distal femoral epiphysis; type 2: the proximal part of the tibia is ossified and visible on radiographs at birth, but the distal tibia absent; type 3: the distal part of the tibia is ossified and visible, but the proximal portion of the tibia is absent. This is the least common type of tibia hemimelia; type 4: the tibia is short, and there is distal tibiofibular diastasis. In these cases, the distal tibial articular surface is absent, there is proximal displacement of the talus, and the tibia and fibula separate at the ankle. Treatment options depends on the type of deformity. Type 1 involves resection of the hypoplastic arm of bifid femur, osteotomy and re-alignment of remaining with knee disarticulation [9]. This was done on the left side in our case. An alternative treatment is a modified brown procedure of centralizing the fibula below the femur, excision of the bifid limb and a syme amputation [5]. However, this operation is only successful if there is an active quadriceps knee extensor mechanism [14]. From literature, Brown procedure uniformly fails in the treatment of Jones type 1a tibial deficiency and it should not be attempted [15].

## Conclusion

Bifid femur with tibia hemimelia is a challenging congenital abnormality whose management remains unclear. Authors recommend tibiofibular synostosis for Jones type 2 tibia hemimelia and knee disarticulation for type 1. However, a more powered study is needed to strengthen the recommendation.

## Competing interests

The authors declare no competing interest.
